# GDF-15 como Biomarcador em Doenças Cardiovasculares

**DOI:** 10.36660/abc.20200426

**Published:** 2021-02-03

**Authors:** Bruna Miers May, Mauricio Pimentel, Leandro Ioschpe Zimerman, Luis Eduardo Rohde

**Affiliations:** 1 Universidade Federal do Rio Grande do Sul Programa de Pós-Graduação em Ciências da Saúde: Cardiologia e Ciências Cardiovasculares Porto AlegreRS Brasil Universidade Federal do Rio Grande do Sul - Programa de Pós-Graduação em Ciências da Saúde: Cardiologia e Ciências Cardiovasculares , Porto Alegre , RS - Brasil; 2 Hospital de Clinicas de Porto Alegre Hospital de Clinicas de Porto Alegre Porto AlegreRS Brasil Hospital de Clinicas de Porto Alegre , Porto Alegre , RS - Brasil

**Keywords:** Doenças Cardiovasculares, Biomarcadores, GDF-15 Fator de Diferenciação de Crescimento, Citocinas, Estresse, Inflamação, Prognóstico

## Abstract

Nos últimos anos, vários biomarcadores estão ganhando importância clínica na avaliação diagnóstica e prognóstica de pacientes com doenças cardiovasculares. O fator de crescimento e diferenciação celular-15 (GDF-15) é uma citocina induzida por estresse e inflamação, membro da família do TGF-, cuja produção no miocárdio foi demonstrada experimentalmente em resposta à injúria isquêmica ou sobrecarga cardíaca. Este novo marcador foi positivamente correlacionado com aumento do risco de eventos cardiovasculares em estudos populacionais e configurou-se preditor independente de mortalidade e prognóstico adverso em pacientes com doença arterial coronariana e insuficiência cardíaca. Este trabalho tem como objetivo revisar o valor diagnóstico e prognóstico do GDF-15 em diferentes cenários na cardiologia.

## Introdução

O fator de crescimento e diferenciação celular-15 (GDF-15) é uma citocina da família do fator de transformação do crescimento beta (TGF-) encontrada em baixa quantidade nos tecidos e no plasma, exceto pela placenta e próstata. Antigamente chamado de citocina inibidora de macrófagos-1 (MIC-1), o GDF-15 foi descoberto há mais de 20 anos e assim nomeado devido a um possível papel antagonista após a ativação de macrófagos por citocinas inflamatórias (interleucinas e fator de necrose tumoral) em estudos experimentais. A sua função no organismo permanece incerta até hoje e pode variar conforme o tecido estudado. A expressão desse marcador é regulada por estresse e injúria tecidual, e está associada a condições inflamatórias em diferentes órgãos, inclusive no miocárdio. ^1^

**Figura 1:**
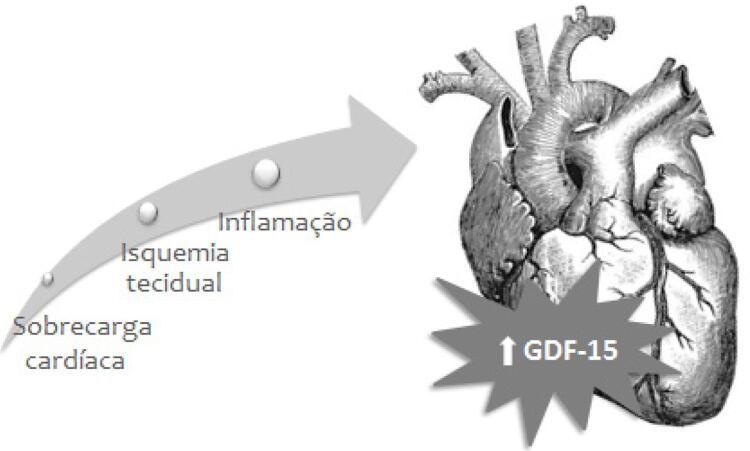
– Fatores que modificam a expressão do fator de crescimento e diferenciação celular-15 (GDF-15) no sistema cardiovascular.

Em modelos animais, o GDF-15 apresentou-se inicialmente como uma proteína cardioprotetora, prevenindo morte celular, e dilatação e hipertrofia cardíaca. Expressão aumentada do marcador foi encontrada após estímulos agressores como sobrecarga de pressão e isquemia tecidual. ^2 , 3^ A ativação da enzima NOS-2 (óxido nítrico sintase 2), em situações de estresse, participa na regulação positiva de GDF-15 por vias de sinalização intracelular dependentes de óxido nítrico. ^3^ Em ratos geneticamente modificados para apresentarem deficiência de GDF-15, na isquemia miocárdica induzida, eram observadas áreas de infarto maiores com maior apoptose de miócitos, indicando uma possível função de limitação de dano miocárdico do marcador. ^3^ A Figura 1 apresenta os principais fatores que influenciam a expressão do GDF-15.

Outro trabalho experimental correlacionou níveis elevados do GDF-15 em cardiomiócitos de ratos com uma redução na ativação do hormônio do crescimento (GH), sugerindo sua participação na via de sinalização do GH. Após essa descoberta, os mesmos autores realizaram estudo em crianças com cardiopatia congênita, encontrando níveis significativamente maiores de GDF-15 no plasma de crianças com cardiopatia e deficiência de crescimento, em relação a controles saudáveis e a cardiopatas com crescimento normal. ^4^

Após os resultados dos trabalhos iniciais, o GDF-15 passou a ser investigado em vários contextos clínicos, apresentando-se na maior parte dos estudos como um biomarcador associado a maior risco de desenvolver eventos cardiovasculares. ^5 - 9^ Atualmente, a dosagem sérica do GDF-15 encontra-se disponível comercialmente apenas na Europa, em outras regiões os kits são acessíveis apenas para fins de pesquisa clínica e experimental. ^10^ A dosagem é realizada por imunoensaios, ensaios por quantificação de complexos antígeno-anticorpo marcados por radioisótopos (técnica de imunorradiometria – IRMA), por enzimas (ELISA) ou luminescentes (quimioluminescência). A faixa de detecção varia entre 400-20000 ng/L, com boa precisão e reprodutibilidade (taxas de imprecisão intra e inter-ensaios abaixo de 10%). O método mais utilizado hoje é o por ELISA, pelo menor custo e maior acessibilidade. ^11 , 12^

O objetivo deste artigo é revisar o papel do GDF-15 em diferentes cenários da cardiologia, avaliando-se a possibilidade de sua incorporação como biomarcardor no diagnóstico e estratificação de risco de cardiopatias prevalentes.

### Risco Cardiovascular em Indivíduos Saudáveis

O primeiro estudo em humanos a relacionar o GDF-15 com doença cardiovascular foi publicado em 2002 e incluiu 27 628 mulheres saudáveis acompanhadas por quatro anos. Os resultados demonstraram um aumento de 2,7 vezes no risco de evoluir com eventos cardiovasculares (infarto, acidente vascular cerebral e morte cardiovascular) nas participantes com níveis da citocina acima de 856 ng/L. ^13^ Em uma coorte com 1391 pacientes sem doença cardiovascular estabelecida, o marcador foi preditor independente de mortalidade por todas as causas e de morte cardiovascular com *hazard ratio* (HR) de 1,5 (IC 95%: 1,3-1,8), com poder discriminatório comparável ao peptídeo natriurético cerebral (BNP) (HR 1,3; IC 95%: 1,2-1,5). ^14^

Dados do Framingham Heart Study, onde foram avaliados 85 biomarcadores (incluindo BNP, PCR e GDF-15) em 3523 participantes ao longo de 14 anos de seguimento, mostraram que o GDF-15 foi o único marcador, em análise multivariada, a manter associação significativa com os três desfechos avaliados: eventos cardíacos ateroscleróticos (HR 1,43; IC 95%: 1,20-1,58), insuficiência cardíaca (IC) (HR 2,08; IC 95%: 1,72-2,53) e mortalidade (HR 1,96; IC 95%: 1,76-2,17). ^8^

### Doença Arterial Coronariana (DAC)

O GDF-15 foi estudado em pacientes admitidos no hospital após síndrome coronariana aguda (SCA) e em portadores de doença coronariana estável.

#### Síndromes Coronarianas Agudas

Pacientes que apresentavam níveis aumentados de GDF-15 em dosagem realizada na internação devido à SCA evoluíram com um número maior de eventos como morte cardiovascular, reinfarto e acidente vascular cerebral em 12 meses de seguimento após a alta, demostrando um valor prognóstico com relação à progressão de doença aterosclerótica. ^15^

Outro estudo observacional recente demonstrou a mesma associação prognóstica do marcador com eventos cardiovasculares maiores (mortalidade total, infarto não fatal e internação por IC). Porém, em análise multivariada ajustada para outros fatores de risco, o GDF-15 permaneceu significativo apenas para mortalidade e desenvolvimento de IC. ^16^

Ainda no contexto de doença aguda, um ensaio clínico avaliando estratégia invasiva versus conservadora na SCA sem supradesnivelamento do segmento ST encontrou incidência significativa mais alta de eventos nos pacientes com níveis elevados de GDF-15 alocados no grupo da estratégia conservadora. Os autores sugerem que a dosagem do marcador possa complementar os escores de risco na seleção daqueles que se beneficiam mais da estratégia invasiva precoce. ^17^

Corroborando com essa ideia, o uso do escore GRACE, ajustado para GDF-15, associado à medida dos níveis de GDF-15 na admissão ao hospital, aumentou a acurácia do escore [área sob a curva (AUC) ROC de 0,79 para 0,85]. Entre os pacientes que não apresentaram eventos durante o seguimento de 6 meses, 54 foram classificados como risco intermediário pelo escore de GRACE e teriam sido reclassificados em baixo risco se utilizado o escore ajustado. ^18^ Tzikas et al., ^19^ encontraram que o GDF-15 é preditor independente de eventos cardiovasculares de modo semelhante à troponina, com forte correlação com a gravidade da doença coronariana avaliada pelo escore Syntax após cateterismo cardíaco. ^19^

Avaliando-se especificamente pacientes com infarto agudo do miocárdio com supradesnivelamento do segmento ST (IAMCSST) tratados com angioplastia primária, a taxa de mortalidade em 10 anos após o evento agudo aumentou de 6% para 19% nos pacientes com GDF-15 acima da mediana. ^20^ Outro estudo avaliou a alteração dinâmica do GDF-15 durante as primeiras 24 horas de um evento de IAMCSST e mostrou pico da citocina em 12 horas, e manutenção dos níveis elevados até final das 24 horas. Níveis maiores na dosagem realizada após 24 horas se correlacionaram à maior mortalidade em 30 dias. ^21^ Em relação à extensão do infarto e prognóstico, quanto maior a dosagem do marcador, maior o risco de remodelamento e dilatação ventricular em 12 meses. ^22^ Estudo prospectivo analisou 92 biomarcadores em 847 pacientes com doença coronariana acompanhados por seis anos após infarto agudo. GDF-15 foi um dos dois únicos marcadores com poder para predizer mortalidade, após ajuste para fatores clínicos. ^23^

Em metanálise incluindo oito estudos com pacientes acompanhados após uma SCA, o GDF-15 foi considerado um forte preditor de mortalidade com risco relativo (RR) de 6,08 (IC 95%: 4,79-7,71; p < 0,001) e reinfarto não-fatal, com RR de 1,76 (IC 95%: 1,49-2,07; p < 0,001). ^24^ Uma metanálise mais recente com 13 estudos e um total de 43 547 pacientes com SCA corrobora esses resultados: RR para mortalidade de 6,75 (IC 95%: 5,81-7,84; p < 0,001) e para reinfarto não-fatal 1,95 (IC 95%: 1,72-2,21, p < 0,001). ^25^

Ainda no cenário de SCAs, em que está indicado o uso de dupla antiagregação plaquetária, o GDF-15 também foi preditor de risco de sangramento. ^15^ Em uma análise post-hoc do ensaio clínico PLATO (ticagrelor x clopidogrel no IAMCSST), foi identificado um risco três vezes mais alto de sangramento nos pacientes que mantém níveis do marcador acima de 1800 ng/L em dosagem realizada um mês após a SCA, independentemente da droga utilizada. ^26^ Nesse contexto, em que os níveis de GDF-15 mantêm-se elevados após o evento agudo, um marcador de risco de sangramento pode ajudar na decisão de manter a terapia antitrombótica dupla além do tempo usualmente recomendado.

#### Doença Coronariana Estável

Na doença coronariana crônica, GDF-15 foi dosado em uma coorte com 14577 pacientes portadores de angina estável, revascularização prévia, e doença multiarterial ou infarto há mais de um ano. Ao longo do seguimento, níveis acima de 1827 ng/L associaram-se a maior risco de morte cardiovascular (HR 2,63; IC 95%: 1,9-3,6; p<0,001), morte súbita cardíaca (HR 3,06; IC 95%: 1,9-4,8; p<0,001) e hospitalização por IC (HR 5,8; IC 95%: 3,2-10; p = 0,006), de forma independente a outros marcadores como troponina, proteína C reativa e BNP. Neste estudo, ^27^ o GDF-15 não se correlacionou com novo evento trombótico após ajuste para os demais biomarcadores.

## Insuficiência Cardíaca (IC)

O GDF-15 foi avaliado em diversas coortes de pacientes portadores de IC e comparado, na maioria, a peptídeos natriuréticos [BNP ou fragmento N-terminal do pró-hormônio do BNP (NT-proBNP)]. A principal diferença entre eles é a proporção do aumento no plasma conforme o tipo de disfunção ventricular. O NT-proBNP, um marcador de estresse hemodinâmico do ventrículo esquerdo, está aumentado na IC com fração de ejeção reduzida de forma mais significativa do que na IC com fração de ejeção preservada. Em contrapartida, o GDF-15 encontra-se elevado de forma semelhante na disfunção sistólica e diastólica, sugerindo que injúria inflamatória seja parte da fisiopatologia de ambas as condições. O GDF-15 apresentou-se como importante preditor de eventos adversos e mortalidade, independentemente da fração de ejeção e nível sérico de NT-proBNP. ^28 - 33^

## IC com Fração de Ejeção Teduzida (ICFEr)

A avaliação do GDF-15 em diferentes estágios da IC define-o como um biomarcador de evolução de doença, aumentando exponencialmente conforme a piora de classe funcional e o remodelamento do ventrículo esquerdo. Os níveis de GDF-15 já estão elevados na fase pré-clínica da IC (estágio B) e a associação do marcador ao NT-proBNP aumentou a acurácia diagnóstica para IC, inclusive nessa fase inicial. ^34^ Na mesma direção, outro estudo prospectivo correlacionou o GDF-15 à progressão da disfunção ventricular e perda de capacidade funcional em pacientes com fração de ejeção menor que 35%, encontrando níveis séricos progressivamente maiores conforme a gravidade da IC. Este resultado se manteve significativo após ajuste para outros fatores de risco como o consumo de oxigênio de pico (VO2 pico), idade e taxa de filtração glomerular. ^35^

O primeiro grande estudo que avaliou o valor prognóstico do GDF-15 na ICFEr foi realizado utilizando-se a base de dados do ensaio clínico Val-HeFT ( *Valsartan Heart Failure Trial* ), que avaliou o uso de valsartan em pacientes com IC. O GDF-15 foi avaliado no início do estudo (n=1734) e após 12 meses de seguimento (n=1517). No início do estudo, 85% dos pacientes apresentavam valores de GDF-15 elevados (>1200ng/ml). Em análise multivariada incluindo variáveis clínicas, BNP, troponina e proteína C reativa, valores elevados de GDF-15 permaneceram associados de forma independente com aumento do risco de mortalidade total (HR 1,007; IC 95%: 1,001-1,014; p=0,02), mas não com a ocorrência de primeiro evento mórbido (HR 1,003; IC 95%: 0,997-1,008; p=0,34), que incluía morte, morte súbita com ressuscitação, hospitalizações por IC, ou necessidade de uso de inotrópicos ou vasodilatadores intravenosos por mais de 4h sem hospitalização. Em 12 meses de seguimento, os valores de GDF-15 aumentaram de modo semelhante no grupo placebo e no grupo valsartan, sendo associados de modo independente com mortalidade total e primeiro evento mórbido. Este achado sugere que o GDF-15 represente um eixo fisiopatológico que não é afetado pelas terapias prescritas. ^7^

Mais recentemente, o GDF-15 foi estudado em 1935 pacientes incluídos no estudo PARADIGM-HF, que comparou sacubitril/valsartana versus enalapril em pacientes com ICFEr. O GDF-15 basal e os seus níveis em um e oito meses de tratamento foram associados significativamente com aumento do risco de mortalidade total e de eventos cardiovasculares. Cada incremento de 20% no GDF-15 basal foi associado com maior risco de mortalidade total (HR 1,13; IC 95%:1,08-1,18; p<0,001), desfecho combinado de morte cardiovascular ou hospitalização por IC (HR 1,09; IC 95%: 1,05-1,14; P<0,001) e morte por IC (HR 1,16; IC 95% 1,05-1,28; p<0,001). O incremento no nível sérico de GDF-15 durante o estudo não foi influenciado pelos tratamentos prescritos. ^36^

O papel do marcador foi estudado ainda em pacientes submetidos a implante de ressincronizador cardíaco. Durante o seguimento de 158 pacientes, 72% apresentaram boa resposta à ressincronização, mas aqueles em que o nível sérico basal do GDF-15 era superior a 2720 ng/L tiveram risco significativamente maior de morte cardiovascular e reinternações por IC em 2,5 anos. Apesar de se demonstrar o valor prognóstico do biomarcador nesta população, o nível basal e a variação em um ano pós-implante não foram capazes de predizer resposta ao dispositivo. ^37^

No cenário de doença avançada, foram dosados cinco biomarcadores (PCR, NT-proBNP, GDF-15, galectina-3 e troponina) em pacientes com classe funcional NYHA (New York Heart Association) III. Entre eles, o GDF-15 foi o melhor preditor de mortalidade a longo prazo, inclusive com maior valor preditivo que NT-proBNP (AUC 0,78 versus 0,63). ^38^

Em pacientes com miocardiopatia não-isquêmica grave, GDF-15 foi analisado em biópsias realizadas durante implante de dispositivos de assistência ventricular ou transplante cardíaco, e se mostrou fortemente correlacionado ao grau de fibrose miocárdica nessas amostras. ^39^ Nesta coorte, observou-se que um mês após o implante do suporte circulatório, os níveis do marcador reduziram-se significativamente comparado ao pré-implante, sugerindo mais uma vez a sua associação com o grau de disfunção miocárdica. ^39^

### IC com Fração de Ejeção Preservada (ICFEp)

Atualmente, os critérios diagnósticos para ICFEp são baseados principalmente em sintomas de IC e alterações ecocardiográficas sugerindo elevação nas pressões de enchimento cardíacas. Ainda assim, há bastante heterogeneidade nos conceitos e critérios adotados pelas Sociedades e no diagnóstico de consultório na prática clínica. Em pacientes com ICFEp, foram detectados níveis elevados de GDF-15, e sua associação direta com a relação E/e’ no ecocardiograma. A combinação de NT-proBNP e GDF-15 elevados aumentou a acurácia diagnóstica (atingindo uma AUC de 0,93) para ICFEp. ^40^ Ainda, estudos de coorte prospectivos com esta população demonstraram que quanto maior o nível sérico do GDF-15, maior o grau de disfunção diastólica e pior a classe funcional NYHA. ^41 , 42^

Um desafio diagnóstico é a definição de ICFEp em obesos mórbidos, devido a limitações ecocardiográficas como janela desfavorável, dispneia multifatorial e níveis reduzidos de BNP. No estudo de Baessler et al., ^43^ em pacientes com índice de massa corporal acima de 30 kg/m ^2^ , o GDF-15 se correlacionou com elevação nas pressões de enchimento ao ecocardiograma. A adição do GDF-15 aos critérios ecocardiográficos de disfunção diastólica obteve melhor performance diagnóstica nessa população, comparada à associação do BNP aos mesmos critérios (AUC 0,76 x AUC 0,56, respectivamente). ^43^

## IC Agudamente Descompensada

A concentração sérica do GDF-15 na admissão de pacientes agudamente descompensados é elevada (a maioria dos estudos encontrou valores acima de 1200 ng/L). Quanto mais alta a concentração do marcador ou se os níveis aumentam durante a internação, maior o risco de reinternações por IC e mortalidade após a alta hospitalar. ^44 , 45^

Estudo ^46^ com 55 pacientes portadores de ICFEr realizou dosagens seriadas de diversos biomarcadores durante a internação por descompensação cardíaca e 30 dias após a alta, demonstrando que a curva do GDF-15 se assemelha à de dois outros marcadores: ST2 (biomarcardor pertencente à família dos receptores de interleucina-1) e BNP. Neste estudo, um rápido decréscimo no nível do marcador é evidenciado com a melhora clínica dos pacientes, diferente do que ocorreu com outras proteínas inflamatórias como proteína C reativa, TNF-alfa, IL-6, galectinas e mieloperoxidase. ^46^

Modelos construídos adicionando o GDF-15 a marcadores clássicos como troponina e BNP demonstram que a sua dosagem na IC aguda acrescenta valor prognóstico. Esse dado sugere a presença de várias vias fisiopatológicas independentes em pacientes hospitalizados por IC e novamente denota relevância clínica do marcador nesse cenário. ^47 , 48^

**Figura 2:**
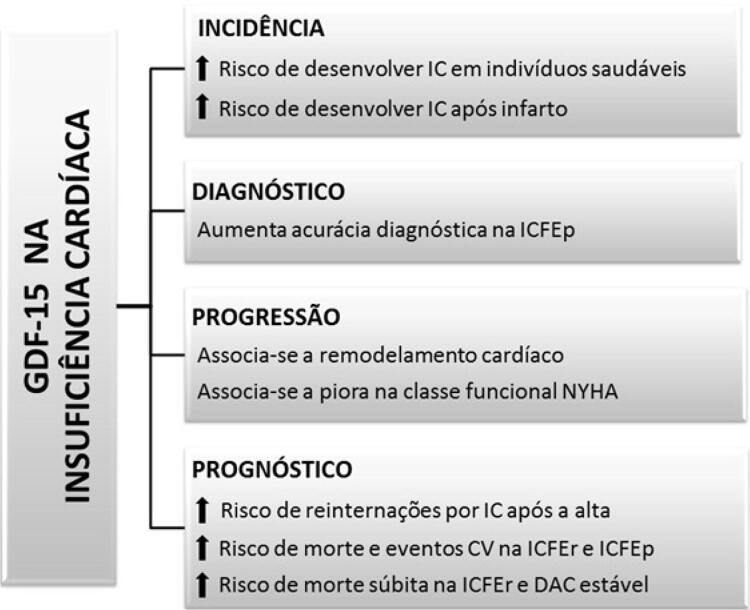
– Implicação do aumento nos níveis de fator de crescimento e diferenciação celular-15 (GDF-15) em diferentes aspectos clínicos na insuficiência cardíaca. IC: insuficiência cardíaca; CV: cardiovasculares; ICFEp: insuficiência cardíaca com fração de ejeção preservada; NYHA: New York Heart Association; ICFEr: insuficiência cardíaca com fração de ejeção reduzida; DAC: doença arterial coronariana

A Figura 2 apresenta as principais correlações do GDF-15 com diferentes aspectos clínicos da IC.

## Morte Súbita

O GDF-15 também foi estudado no contexto de estratificação de risco de morte súbita em pacientes com doenças cardiovasculares. No cenário de doença coronariana estável, demonstrou-se que pacientes com GDF-15 elevado apresentam maior risco de morte súbita com razão de risco ( *hazard ratio* ) de 3,0 (IC 95%: 1,94-4,84; p < 0,001). ^27^

Em um estudo coorte recente, realizou-se a dosagem de ST2 e GDF-15 em 52 pacientes portadores de IC de etiologia não isquêmica, acompanhados por um tempo médio de sete anos. O GDF-15 correlacionou-se com um aumento em duas vezes no risco de morte por arritmia e morte súbita com ressuscitação (HR 2,2; IC 95% 1,1-4,5; p=0,028) e foi superior ao ST2 em predizer mortalidade por qualquer causa (HR 2,4; IC 95%: 1,4-4,2; p = 0,003 *versus* HR 1,6; IC 95%: 1,05-2,7; p = 0,03). ^49^

## Fibrilação Atrial (FA)

Em pacientes com FA, em tratamento adequado e anticoagulados, aqueles com níveis mais elevados do marcador apresentaram taxas 4 a 5 vezes mais altas de mortalidade, independentemente da idade, sexo e escore CHA _2_ DS _2_ VASc. ^50^ Resultado semelhante foi encontrado por Sharma et al., ^51^ sendo o GDF-15 fortemente associado à morte por progressão da IC e sangramento. ^51^

Pacientes portadores de FA não valvar, não anticoagulados, cujos níveis de GDF-15 sérico são acima de 809 ng/dL têm mais risco de ter trombo no átrio esquerdo, independente de idade, volume do átrio e CHA _2_ DS _2_ VASc. ^52^

Em um estudo com 14.798 pacientes anticoagulados, foi encontrado um risco 3,5 vezes mais elevado de sangramento maior nos participantes com GDF-15 elevado, independente da terapia antitrombótica utilizada e demais comorbidades. ^50^ Após esse achado, foi desenvolvido nesta população e validado em outra amostra semelhante o escore de risco ABC (baseado em idade, biomarcadores - GDF-15, hemoglobina e troponina - e história clínica de sangramento), para sangramentos, sendo o GDF-15 o integrante de maior contribuição para o risco. Esse escore obteve melhor acurácia que o escore mais utilizado na prática clínica (HAS-BLED). ^53^

## Doença Renal Crônica

Remodelamento cardíaco, fibrose e inflamação são possíveis fatores envolvidos no aumento da incidência de eventos cardiovasculares em pacientes com doença renal crônica (DRC).

Avaliando-se biomarcadores possivelmente representativos dessas condições, demonstrou-se que as proteínas ST2, galectina-3 e GDF-15 associaram-se significativamente à mortalidade nestes pacientes, mas não a eventos ateroscleróticos. Entre esses, apenas o GDF-15 correlacionou-se ao risco de desenvolver IC. ^54^

Resultado semelhante foi encontrado por Bansal et al., ^55^ em que o GDF-15 foi preditor de IC em pacientes com disfunção renal, assim como o NT-proBNP. Entretanto, ao contrário do peptídeo natriurético, o GDF-15 teve uma correlação mais forte com ICFEp. ^55^

Pacientes com taxa de filtração glomerular abaixo de 60 mL/min/1,73m ^2^ apresentam níveis significativamente maiores de GDF-15 e NT-proBNP, quando comparados a pacientes com função renal normal. Em uma coorte com 358 pacientes com DRC e disfunção sistólica, o GDF-15 refinou a estratificação prognóstica de pacientes com NT-proBNP baixo e foi fortemente associado a eventos adversos, de maneira mais significativa que o próprio peptídeo. ^56^

Na Tabela 1 encontram-se os valores, em média, dos níveis séricos de GDF-15 associados às condições clínicas exploradas nesta revisão.

**Tabela 1:** Pontos de corte do fator de crescimento e diferenciação celular-15 (GDF-15) empregados no diagnóstico e prognóstico em diferentes condições clínicas (valores expressos em ng/L)

Valor para diagnóstico		Predição de eventos adversos			
		Eventos cardiovasculares	Morte cardiovascular	Morte súbita	Mortalidade total
IC com fração de ejeção reduzida ^7,33,34^	> 1200	> 2040	> 2252	*	> 2040
IC com fração de ejeção preservada ^31,40,42^	> 1160	*	NA	NA	*
Síndrome coronariana aguda ^15,19^	> 967	> 1550	> 1550	NA	> 1259
Doença arterial coronariana estável ^27^	NA	> 1253	> 1827	> 1253	> 915

NA: Não avaliável (sem estudos para aquele desfecho ou estudos pequenos não confiáveis). * Uso do biomarcador como variável contínua, sem ponto de corte específico.

## Conclusão

O GDF-15 é um biomarcador sérico cuja expressão parece ser afetada por estresse, injúria tecidual e inflamação, embora seu eixo fisiopatológico ainda não seja completamente entendido. Estudos observacionais com pessoas saudáveis demonstraram associação do GDF-15 com maior risco de eventos cardiovasculares ao longo do tempo. Em pacientes com doença arterial coronariana e IC, o GDF-15 correlacionou-se a um risco elevado de mortalidade total e eventos adversos. A incorporação do GDF-15 melhorou a performance diagnóstica da ICFEp e contribuiu para a elaboração de escore mais acurado para risco de sangramento na FA. A utilização do GDF-15 na prática clínica como marcador prognóstico e sua capacidade de orientar a tomada de decisão clínica depende ainda de novos estudos com maior número de pacientes.
