# Supplementary material for: GDF-15 como Biomarcador em Doenças Cardiovasculares
Source: Arq Bras Cardiol. 2021 Feb 3;116(3):494–500. [Article in Portuguese] doi: 10.36660/abc.20200426 (PMC8159541; doi:10.36660/abc.20200426)
Supplement: Translation

# Translation: GDF-15 as a Biomarker in Cardiovascular Disease

**Authors:** Bruna Miers May, Mauricio Pimentel, Leandro Ioschpe Zimerman, Luis Eduardo Rohde

**Affiliations:** 1 Brazil Universidade Federal do Rio Grande do Sul - Programa de Pós-Graduação em Ciências da Saúde: Cardiologia e Ciências Cardiovasculares, Porto Alegre, RS - Brazil; 2 Brazil Hospital de Clinicas de Porto Alegre, Porto Alegre, RS - Brazil

**Keywords:** Cardiovascular Diseases, Biomarkers, GDF-15 Growth Differentiation Factor 15, Cytokines, Stress, Inflammation, Prognostic

## Abstract

In the last years, several diagnostic and prognostic biomarkers have been studied in cardiovascular disease. Growth differentiation factor-15 (GDF-15), a cytokine belonging to the transforming growth factor- (TGF-) family, is highly up-regulated in stress and inflammatory conditions and has been correlated to myocardial injury and pressure cardiac overload in animal models. This new biomarker has been positively correlated with increased risk of cardiovascular events in population studies and shown an independent predictor of mortality in patients with coronary artery disease and heart failure. This review aimed to summarize the current evidence on the diagnostic and prognostic value of GDF-15 in different settings in cardiology.

## Introduction

The growth differentiation factor-15 (GDF-15) is a cytokine belonging to the transforming growth factor beta (TGF-ß) family, with low concentrations in tissue and plasma, except for the placenta and prostate. GDF-15, discovered more than 20 years ago, was formerly named the macrophage inhibitory cytocine-1 (MIC-1) due to its possible role as an antagonist of macrophage activation by inflammatory cytokines (interleukins and tumor necrosis factor) in experimental studies. The role of this cytokine in human body has not been elucidated yet and seems to vary with tissue types. The expression of this marker is upregulated by stress and tissue damage, and is associated with inflammatory conditions of different organs, including the myocardium.^1^

In animal models, the GDF-15 was initially reported as a cardioprotective protein, preventing cell death, and cardiac dilatation and hypertrophy. An increased expression of this marker was seen in response to damaging stimuli, such as pressure overload and tissue ischemia.^2 , 3^The activation of nitric oxide synthase (NOS-2) enzyme in stressful situations is involved in the up-regulation of GDF-15 via intracellular signaling pathways, depending on nitric oxide.^3^In genetically modified rats deficient in GDF-15, greater infarction areas with myocyte apoptosis were detected in induced myocardial infarction, suggesting a protective role of GDF-15 against myocardial injury.^3^Figure 3 presents the main factors that influence the GDF-15 expression.

Another experimental study correlated the increased levels of GDF-15 in cardiomyocytes of rats with reduced activation of growth hormone (GH), suggesting the involvement of this marker in GH signaling pathway. After this finding, the same authors conducted a study on children with congenital heart disease, and found significantly higher levels of GDH-15 in plasma of children with concomitant heart disease and failure to thrive compared with healthy controls and children with heart disease and normal growth.^4^

Since then, the GDF-15 has been investigated in several clinical conditions, and associated with a greater risk for cardiovascular events in most of the studies.^5 - 9^Today, the kits for determination of serum GDF-15 levels can be found commercially available in Europe, while in other regions, they are used for research and experimental purposes only.^10^The measurement is made by immunoassays, by immunoradiometric assay (IRMA) that determines the amount of the radiolabeled antigen–antibody complex, by enzymes (ELISA) or by luminescence (chemiluminescence). The detection range varies from 400 to 20000 ng/L, with good precision and reproducibility (within-and between-assay imprecision lower than 10%). The most used test today is the ELISA due to is lower cost and higher accessibility.^11 , 12^

The aim of this article is to review the role of GDF-15 in different cardiac diseases and evaluate the possibility of incorporating it as a biomarker in the diagnosis and risk stratification of common heart diseases.

### Cardiovascular Risk in Healthy Individuals

The first human study to correlate GDF-15 with cardiovascular disease was published in 2002 and included 27,628 healthy women, followed-up for four years. The results indicated a 2.7-fold increase in the risk of developing cardiovascular events in women with GDF-15 concentrations above 856 ng/L.^13^In a cohort of 1,391 patients with no history of heart disease, GDF-15 was an independent predictor of mortality and cardiovascular mortality, with a hazard ratio (HR) of 1.5 (95% CI 1.3-1.8), with a discriminatory power comparable to B-type natriuretic peptide (BNP) (HR 1.3; 95% CI 1.2-1.5).^14^

Data from the Framingham Heart study, in which 85 biomarkers were evaluated (including BNP, PCR and GDF-15) in 3,523 participants over 14 years, showed that the GDF-15 was the only marker, in a multivariate analysis, to show a significant association with the three outcome results: atherosclerotic events (HR 1.43; 95%CI: 1.20-1.58), heart failure (HF) (HR 2.08; 95%CI: 1.72-2.53) and mortality (HR 1.96; 95%CI: 1.76-2.17).^8^

### Coronary Artery Disease (CAD)

GDF-15 was studied in patients admitted for acute coronary syndrome (ACS) and patients with stable coronary disease.

#### Acute Coronary Syndromes

Patients with elevated GDF-15 at admission for ACS had more events such as cardiovascular deaths, reinfarction and stroke at 12 months after discharge, indicating a prognostic value regarding the course of atherosclerotic disease.^15^

Another recent observational study showed the same prognostic association of GDF-15 with major cardiovascular events (overall mortality, non-fatal infarction, and hospitalization for HF). However, in a multivariate analysis adjusted for other risk factors, the GDF-15 remained significant only for mortality and development of HF.^16^

Still in the context of acute diseases, a clinical trial comparing invasive versus conservative strategy in non-ST-elevation acute coronary syndrome showed a significantly higher incidence of events in patients with elevated GDF-15 levels, allocated to the conservative strategy group. The authors suggested that the determination of GDF-15 should complement risk scores in the screening for patients who would benefit more of an early invasive strategy.^17^

In agreement with this idea, the use of the GRACE risk score, adjusted for GDF-15, combined with GDF-15 measurement at hospital admission increased the score accuracy (area under the receiver-operating characteristic curve from 0.79 to 0.85). During the six-month follow-up, 54 of the patients without events were classified into low risk according to the adjusted score.^18^Tzikas et al.^19^found that GDF-15 is an independent predictor of cardiovascular events, comparable to troponin. Also, the authors observed that GDF-15 levels were strongly correlated with the severity of coronary disease assessed by the Syntax score after coronary revascularization.^19^

In a study on patients with ST-segment elevation myocardial infarction treated with primary percutaneous coronary intervention, the10-year all-cause mortality rate following an acute event increased from 6% to 19% in patients GDF-15 levels above the median.^20^Another study evaluated GDF-15 temporal dynamics during the first 24 hours of ST-elevation myocardial infarction and showed that GDF-15 peaked at 12 h and remained elevated at 24 h. At 24 hours, higher levels of GDF-15 were correlated with higher 30-day mortality.^21^With respect to the extension of infarction and prognosis, the higher the GDF-15 levels, the greater the risk for ventricular remodeling and dilation in 12 months.^22^A prospective study analyzed 92 biomarkers in 847 patients with coronary disease followed for six years after acute infarction. GDF-15 was one of the markers with a predictive power for mortality after adjustments for clinical factors.^23^

A meta-analysis of eight studies with STEMI patients, the GDF-15 was considered a strong predictor of mortality, with a relative risk (RR) of 6.08 (95%CI: 4.79-7.71; p < 0.001) and of non-fatal reinfarction, with a RR of 1.76 (95%CI: 1.49-2.07; p < 0.001).^24^These findings are corroborated in a more recent meta-analysis of 13 studies and 43,547 patients with ACS: RR for mortality of 6.75 (95%CI: 5.81-7.84; p < 0.001) and RR for non-fatal reinfarction of 1.95 (95%CI: 1.72-2.21, p < 0.001).^25^

In addition, for ACS patients with indication for dual antiplatelet therapy, GDF-15 was a predictor of bleeding risk.^15^In a post-hoc analysis of the PLATO trial (ticagrelor vs. clopidogrel in STEMI), patients with elevated GDF-15 (>1800 ng/L) at one month after an ACS was associated with a three-fold increased risk of bleeding, regardless of the drug used.^26^In this case of elevated GDF-15 levels after an acute event, a marker of bleeding risk may help in the decision to continue the dual antithrombotic therapy beyond the usual time.

## Stable Coronary Disease

In chronic coronary disease, GDF-15 was measured in a cohort of 14,577 patients with stable angina and history of revascularization, multivessel disease or infarction for more than one year. During the follow-up period, GDF-15 levels above 1,827ng/L were associated with increased risk of cardiovascular death (HR 2.63; 95%CI 1.9-3.6; p<0.001), cardiac sudden death (HR 3.06; 95%CI: 1.9-4.8; p<0.001) and hospitalization for HF (HR 5.8; 95%CI: 3.2-10.0; p = 0.006), regardless of other markers such as troponin, reactive C protein, and BNP. In this study,^27^no correlation was found between GDF-15 and new thrombotic event after adjustment for the other biomarkers.

## Heart Failure

GDF-15 was assessed in different cohorts of patients with HF, and compared with natriuretic peptides [BNP or the N-terminal fragment of BNP precursor (NT-proBNP)]. The main difference between them was the magnitude of increase in plasma concentrations according to the type of ventricular dysfunction. NT-proBNP, a marker for hemodynamic stress on the left ventricle was more significantly increased in HF with reduced ejection fraction than in HF with preserved ejection fraction. On the other hand. GDF-15 was similarly increased in both systolic and diastolic dysfunction, suggesting that the inflammatory injury is involved in the pathophysiology of both conditions. The GDF-15 was shown to be an important predictor of adverse events and mortality, independent of ejection fraction and serum levels of NT-proBNP.^28 - 33^

## HF with Reduced Ejection Fraction (HFrEF)

The assessment of GDF-15 in different stages of HF has revealed that the cytokine is a biomarker of disease progression, which increases exponentially with worsening of functional class and remodeling of the left ventricle. The GDF-15 levels are already elevated in the preclinical stage of HF (stage B) and its combination with NT-proBNP increased the diagnostic accuracy for HF, including at this initial stage.^34^In the same line of thought, another prospective study correlated GDF-15 with ventricular dysfunction progression and loss of functional capacity in patients with ejection fraction below 35%, and showed that GDF-15 levels increased with the severity of HF. This result remained significant after adjustment for other risk factors such as peak oxygen consumption (VO2 peak), age and glomerular filtration rate.^35^

The first large study that evaluated the prognostic value of GDF-15 in HFrEF was conducted with data from the Valsartan Heart Failure Trial (Val-HeFT) that evaluated the use of valsartan in HF patients. GDF-15 levels were evaluated in the beginning of the study (n=1,734) and at 12 months of follow-up (n=1,517). In the beginning of the study, 85% of patients had increased GDF-15 levels (>1,200 ng/mL). In a multivariate analysis including clinical variables, BNP, troponin and C-reactive protein, elevated levels of GDF-15 were independently associated with an increase in the risk of overall mortality (HR 1.007; 95%CI: 1.001-1.014; p=0.02), but not with the occurrence of the first morbid event (HR 1.003; 95%CI: 0.997-1.008; p=0.34), that included death, sudden death with resuscitation, hospitalization for HF, or administration of intravenous inotropic or vasodilator drugs for more than 4 hours without hospitalization. After 12 months of follow-up, the increase in the GDF-15 values was similar for the placebo and the valsartan groups and was independently associated with overall mortality and first morbid event. This result suggests that the GDF-15 represents a pathophysiological axis that is not addressed by the therapies prescribed.^7^

More recently, the GDF-15 was studied in 1,935 patients included in the PAADIGM-HF study, which compared sacubitril/valsartan versus enalapril in patients with HFrEF. Baseline GDF-15 and the levels at one month and eight months of treatment were associated with increased risk of overall mortality and cardiovascular events (HR 1.13; 95%CI: 1.08-1.18; p<0.001), combined endpoint of cardiovascular death or hospitalization for HF (HR 1.09, 95% CI 1.05-1.14, p < 0.001) and HF death. The increment in GDF-15 levels was not influenced by the therapies.^36^

The role of GDF-15 was also evaluated in patients undergoing cardiac resynchronization therapy. Of 158 patients, 72% had a good response to treatment; however, patients with serum GDF-15 above 2,720 ng/L had significantly higher risk of cardiovascular mortality, and rehospitalization for HF in 2.5 years. Despite the prognostic value of GDF-15 in this population, baseline levels and changes from baseline one year after implantation failed to predict the response to the resynchronization device.^37^

In advanced HF, five biomarkers (PCR, NT-proBNP, GDF-15, galectin-3, and troponin) were measured in patients New York Heart Association class III. Among these, GDF-15 was the marker that best predicted long-term mortality, with better predictive value as compared with NT-proBNP (area under the curve [AUC] 0.78 versus 0.63).^38^

In patients with severe non-ischemic cardiomyopathy, GDF-15 was evaluated in myocardial tissue obtained during implantation of left ventricular assist devices or during heart transplantation, and found to be strongly correlated with the severity of myocardial fibrosis.^39^In this cohort, at one month after implantation of mechanical circulatory support, GDF-15 levels were significantly decreased compared with pre-implantation levels, which reinforces its association with the severity of myocardial dysfunction.^39^

## HF with Preserved Ejection Fraction (HFpEF)

Today, the diagnostic criteria for HFpEF are mainly based on HF symptoms and echocardiographic changes suggesting elevated cardiac filling pressures. However, there is still high heterogeneity of concepts and criteria adopted by the Societies and in the diagnosis in clinical practice. In HFpEF patients, elevated GDF-15 were detected, with a direct association with echocardiographic E/e ratio. The combination of NT-proBNP with elevated GDF-15 increased the diagnostic accuracy (AUC of 0.93) for HFpEF.^40^Also, prospective cohort studies with this population showed that the higher the GDF-15 levels, the more severe the diastolic dysfunction and NYHA functional class.^41 , 42^

One diagnostic challenge is the definition of HFpEF in morbidly obese patients, due to echocardiographic limitations such as unfavorable window, multifactorial dyspnea, and reduced BNP levels. In the study by Baessler et al.,^43^on patients with body mass index above 30 kg/m^2^, GDF-15 correlated with increased filling pressures at echocardiography. The inclusion of the GDF-15 in the echocardiographic criteria for diastolic dysfunction yielded better diagnostic performance in this population, compared with the combination of BNP with the same criteria (AUC 0.76 x AUC 0.56, respectively).^43^

### Acutely Decompensated HF

Serum GDF-15 concentrations of patients with acutely decompensated HF are elevated at admission (most studies have reported GDF-15 levels above 1,200 ng/L). The higher the GDF-15 concentrations, or if GDF-15 levels increased during hospitalization, the greater the risk of rehospitalizations for HF and post-discharge mortality.^44 , 45^

In a study^46^with 55 patients with HFrEF, the authors conducted serial measurements of several biomarkers during hospitalization for cardiac decompensation and at 30 days of discharge, and showed that the curve pf GDF-15 was similar to two other markers: suppression of tumorigenicity 2 (ST2, a biomarker belonging to the interleukin-1 receptor family) and BNP. In this study, the rapid fall in GDF-15 levels was marked by an evident clinical improvement of patients, different to what was observed with other pro-inflammatory proteins, including C-reactive protein, TNF-alpha, IL-6, galectins and myeloperoxidase.^46^

Models combining the GDF-15 with classical biomarkers such as troponin and BNP have demonstrated that the measurement of this cytokine in acute HF adds prognostic value. This data suggests that the presence of several independent pathophysiological pathways in patients hospitalized for HF, and indicates, once again, the clinical relevance of this biomarker in this scenario.^47 , 48^

Figure 2 shows the main correlations of GDF-15 with clinical aspects in HF.

**Figure 2:** – Implications of increased growth differentiation factor-15 (GDF-15) levels in different clinical conditions of heart failure; HF: heart failure; HFpEF: heart failure with preserved ejection fraction; HFrEF: heart failure with reduced ejection fraction; NYHA: New York Heart Association; CAD: coronary artery disease

## Sudden Death

GDF-15 was also studied in risk stratification for sudden death in patients with cardiovascular diseases. Patients with stable coronary disease and elevated GDF-15 had increased risk for sudden death (HR 3.0) (95%CI: 1.94-4.84; p < 0.001).^27^

In a recent cohort study, measurements of ST2 and GDF-15 levels were determined in 52 nonischemic HF patients followed for a mean of seven years. GDF-15 was correlated with a two-fold increased risk for death for arrhythmia and sudden death with resuscitation (HR 2.2; 95%CI 1.1-4.5; p=0.028) and was superior to ST2 in predicting all-cause mortality (HR 2.4; 95%CI: 1.4-4.2; p = 0.003 *versus* HR 1.6; 95%CI: 1.05-2.7; p = 0.03).^49^

## Atrial Fibrillation (AF)

Among patients with atrial fibrillation, receiving adequate treatment and anticoagulation, those with elevated GDF-15 had four to five-fold higher mortality rate, independently of age, sex and CHA_2_DS_2_VASc.^50^A similar finding was seen in the study by Sharma et al.,^51^where GDF-15 was strongly associated with death due to HF and bleeding.^51^

Nonanticoagulated nonvalvular AF patients with serum levels of GDF-15 above 809 ng/dL are at higher risk for developing left atrial thrombus, regardless of age, atrial volume, and CHA_2_DS_2_VASc.^52^

In a study with 14,798 anticoagulated patients, those with elevated GDF-15 levels had a 3.5-fold increased risk of major bleeding, regardless of the antithrombotic therapy and other comorbidities.^50^After this finding, the ABC (age, biomarkers [GDF-15, hemoglobin and troponin], and clinical history)-bleeding risk score was developed and validated, and the GDF-15 was the biomarker that most contributed to the risk. The ABC bleeding score showed better accuracy than the HAS-BLED score, which is the most used score in clinical practice.^53^

## Chronic Renal Disease

Cardiac remodeling, fibrosis, and inflammation are possibly involved in the increase of cardiovascular events in patients with chronic renal disease (CRD).

Analysis of biomarkers possible representative of these conditions, the ST2, galectin-3 and GDF-15 were found to be significantly associated with mortality in these patients, but not with atherosclerotic events. Among these, only GDF-15 correlated with the risk of developing HF.^54^

Similar results were found by Bansal et al.,^55^who reported that GDF-15 was a predictor of HF in patients with renal dysfunction, similarly to NT-proBNP. However, unlike the natriuretic peptide, the GDF-15 was more strongly correlated with HFpEF.^55^

Patients with a glomerular filtration rate below 60 mL/min/1.73m^2^showed significantly higher levels of GDF-15 and NT-proBNP as compared with patients with normal renal function. In a cohort of 358 patients with CRD and systolic dysfunction, GDF-15 refined the prognostic stratification of patients with low NT-proBNP and was more strongly associated with adverse events than the peptide itself.^56^

Table 1 describes serum GDF-15 levels (mean) associated with the clinical conditions addressed in this review.

**Table 1:** – Cut-off points of growth differentiation factor-15 (GDF-15) used for diagnosis and prognosis in different clinical conditions (values in ng/L)

Diagnostic cut-off levels		Prediction of adverse events			
		Cardiovascular evens	Cardiovascular death	Sudden death	Overall mortality
HF with reduced ejection fraction^7,33,34^	> 1,200	> 2,040	> 2,252	*	> 2,040
HF with preserved ejection fraction^31,40,42^	> 1,160	*	NA	*NA*	*
Acute coronary syndrome^15,19^	> 967	> 1,550	> 1,550	NA	> 1,259
Stable coronary artery disease^27^	NA	> 1,253	> 1,827	> 1,253	> 915

NA: Not accessible (no study including the outcome, or unreliable, small studies). * Use of growth differentiation factor-15 (GDF-15) as a continuous variable, without specific cut-off point.

## Conclusion

GDF-15 is a serum biomarker whose expression seems to be affected by stress, tissue damage, and inflammation, although its pathophysiological pathway has not been elucidated yet. Observational studies with healthy individuals have shown an association of GDF-15 with higher risk of cardiovascular events over time. In patients with coronary artery disease and HF, GDF-15 was correlated with increased risk of overall mortality and adverse events. The use of GDF-15 improved the diagnostic performance for detecting HFpEF and contributed to the development of a more accurate risk score to predict bleeding in AF. The use of GDF-15 as a prognostic marker in clinical practice and its capacity to guide the decision-making process still depends on new studies with larger samples.

**Figure 1:** – Influencing factors of growth differentiation factor-15 (GDF-15) in the cardiovascular system.
